# Feasibility of Remote Intensive Monitoring: A Novel Approach to Reduce Black Postpartum Maternal Cardiovascular Complications

**DOI:** 10.1111/jmwh.13743

**Published:** 2025-02-26

**Authors:** Michelle Villegas‐Downs, Tara A. Peters, Jared Matthews, Anne M. Fink, Alicia K. Matthews, Judith Schlaeger, Aiguo Han, William D. O'Brien, Joan E. Briller, Woon‐Hong Yeo, Barbara L. McFarlin

**Affiliations:** ^1^ Department of Human Development Nursing Science University of Illinois Chicago Chicago Illinois; ^2^ IEN Center for Human‐Centric Interfaces and Engineering at the Institute for Electronics and Nanotechnology Georgia Institute of Technology Atlanta Georgia; ^3^ Department of Biobehavioral Nursing Science University of Illinois Chicago Chicago Illinois; ^4^ Department of Behavioral Sciences Columbia University School of Nursing New York New York; ^5^ Department of Biomedical Engineering and Mechanics Virginia Polytechnic Institute and State University Blacksburg Virginia; ^6^ Department of Electrical and Computer Engineering University of Illinois Urbana‐Champaign Urbana Illinois; ^7^ Division of Cardiology Department of Medicine Department of Obstetrics and Gynecology University of Illinois Chicago Chicago Illinois; ^8^ George W. Woodruff School of Mechanical Engineering Georgia Institute of Technology Atlanta Georgia

**Keywords:** Black postpartum women, heart rate variability, postpartum, remote monitoring, wearable physiologic sensors

## Abstract

**Introduction:**

Approximately 53% of maternal mortality occurs in the postpartum period, a time with little monitoring and health surveillance. The objective of this study was to test the feasibility, usability, appropriateness, and acceptability of remote low‐burden physiologic monitoring of Black postpartum women, using a novel soft wearable patch and home vital sign monitoring for the first 4 weeks postpartum.

**Methods:**

A prospective longitudinal cohort feasibility study of 20 Black postpartum women was conducted using home monitoring equipment and a wearable patch with physiologic sensors measuring temperature, pulse oximetry, blood pressure, electrocardiogram (ECG), heart rate, and respiration twice daily during the first 4 weeks postpartum. Feasibility, acceptability, appropriateness, and usability were measured at the end of the study with the Feasibility of Intervention Measure, Acceptability of Intervention Measure, Intervention Appropriateness Measure, and System Usability Scale.

**Results:**

Twenty Black women were recruited and consented to participate in the study. Remote physiologic monitoring using a wearable patch and home monitoring equipment was rated as feasible (93%), acceptable (93%), appropriate (92%), and useable (80%). During the first 2 weeks postpartum, remote home monitoring detected that 60% of the women had blood pressures exceeding 140/90 mm Hg. The wearable patch provided useable data on ECG, heart rate, heart rate variability, pulse oximetry, and temperature.

**Discussion:**

Our research suggests that remote monitoring in the first 4 weeks postpartum has the potential to identify Black women at risk for postpartum complications.

## INTRODUCTION

A woman dying during pregnancy or after childbirth is not supposed to happen. It is a tragedy for the woman, family, and society.^1^ The US maternal mortality rate, defined as the number of maternal deaths per 100,000 live births during pregnancy and the first year after birth, has increased from 7.2 in 1987 to 19.0 per 100,000 in 2023.^2^ The actual number of maternal deaths may be higher due to difficulties in maintaining contact with women once they are discharged from the hospital. Alarmingly, the overall maternal mortality rate among non‐Hispanic Black women was 51.1 per 100,000, compared with 14.7 for non‐Hispanic White women in 2023.^2^ For every maternal death in the United States, at least another 100 women suffer from severe pregnancy or postpartum morbidities such as infection, heart failure, cardiomyopathy, myocardial infarction, or hypertensive crisis that can have lifelong effects on their health and quality of life.^1^, ^3^, ^4^, ^5^ Despite efforts to reduce complications leading to maternal mortality among Black women, the rates remain unacceptably high.

**QUICK POINTS**

✦ Remote home monitoring with wearable sensors and home monitoring equipment was rated by participants as feasible, acceptable, appropriate, and useable.
✦ Heart rate variability, although not used in routine clinical care, may be a biomarker to identify women at risk for cardiovascular complications.
✦ Remote monitoring in the first 4 weeks postbirth has the potential to improve postpartum care for Black women.

Examining maternal mortality trends during the postpartum period, the time with the least amount of health surveillance, reveals women are most at risk for developing adverse cardiovascular events or death.^1^ Approximately 53% of maternal mortality occurs in the postpartum period.^6^ A recent guideline from the American College of Obstetricians and Gynecologists^7^ recommended interventions to reduce maternal morbidity and mortality, such as identifying women with underlying cardiac disease, hypertension, diabetes, and obesity for increased and targeted surveillance during pregnancy and postpartum. Most interventions have focused on identifying and ameliorating mortality risk factors and adding more outpatient office visits. However, during the postpartum period, women are sleep‐deprived, fatigued, and often have difficulty keeping scheduled and additional health care appointments.^8^

There is evidence that intensive physiologic monitoring may reduce maternal morbidity and mortality during the intrapartum period, especially for Black women.^1^ Analysis of a data set from 2002 to 2014 found a significant annual reduction in mortality during hospitalization in the intrapartum period when women are routinely monitored. Among Black women, mortality was reduced by 4.7% (95% CI, 1.3%‐8%) compared with 3.3% (95% CI, 0.5%‐6%) for White women.^1^ Because intensive monitoring of Black women in the intrapartum period significantly reduced mortality, it is reasonable to investigate if intensive physiologic monitoring in the postpartum period could yield similar reductions in maternal morbidity and mortality. During the intrapartum period, continuous fetal monitoring, maternal blood pressure (BP) measurement, pulse oximetry, and resuscitative care are routinely available and used for all hospital births. Similarly, remote wearable monitoring is being used for many health and illness conditions, including devices that assess daily physiologic stress,^9^ electrocardiograms (ECGs) for individuals with cardiac disease,^8^ face masks to monitor sleepiness,^10^ and newborn pacifiers that monitor electrolytes.^11^ Physiologic changes that occur during the postpartum period may alter the sympathetic and parasympathetic regulation of heart rate (HR). Improved knowledge about HR as a measured quantity (hereafter, HR variability [HRV]) and cardiovascular health after childbirth could enhance future vital sign monitoring by providing a detailed assessment of beat‐to‐beat fluctuations in HR.^12^ Although HRV is monitored to evaluate fetal tolerance of labor, there have been limited reports of its utility in women during and after pregnancy.^13^, ^14^, ^15^ Thus, the potential for remote monitoring for a range of health conditions is possible.^16^, ^17^

Building on prior maternal mortality and cardiovascular research,^1^, ^3^, ^4^, ^5^, ^13^, ^14^, ^15^ we aim to determine whether intensive remote home monitoring during the postpartum period may also decrease maternal morbidity and mortality. The objective of this study was to test the feasibility of low‐burden remote physiologic monitoring in the postpartum period in low‐risk and high‐risk Black women, using a novel soft wearable patch along with home monitoring equipment.

## METHODS

We conducted a prospective longitudinal cohort feasibility study using purposive sampling of Black pregnant women. Our research was approved by the Institutional Review Board (IRB) at the University of Illinois Chicago. Written informed consent was obtained at enrollment and again verbally after birth. Reflexivity was carefully assessed during the study. The 4 researchers who had direct contact with participants (M.V.D., T.A.P., A.K.M., and B.L.M.) included 2 nurses, 1 clinical psychologist, and 1 clinical coordinator. Two researchers identify as people of color, and the other 2 identify as White. The researchers actively reflected on their positionalities and considered how their backgrounds and identities might influence their interactions with participants and the interpretation of data throughout the study. The Strengthening the Reporting of Observational Studies in Epidemiology^18^ checklist was used and is provided in the Supporting Information: Appendix S1.

### Sample and Setting

Our goal was to recruit a sample of 20 Black pregnant women. Women were recruited from the Women's Clinic at the University of Illinois Chicago from January to August 2022. All participants self‐identified as Black and female at birth and at the time of study enrollment. A requirement for enrollment was having access to Wi‐Fi at home.

### Procedures

**Figure 1:**
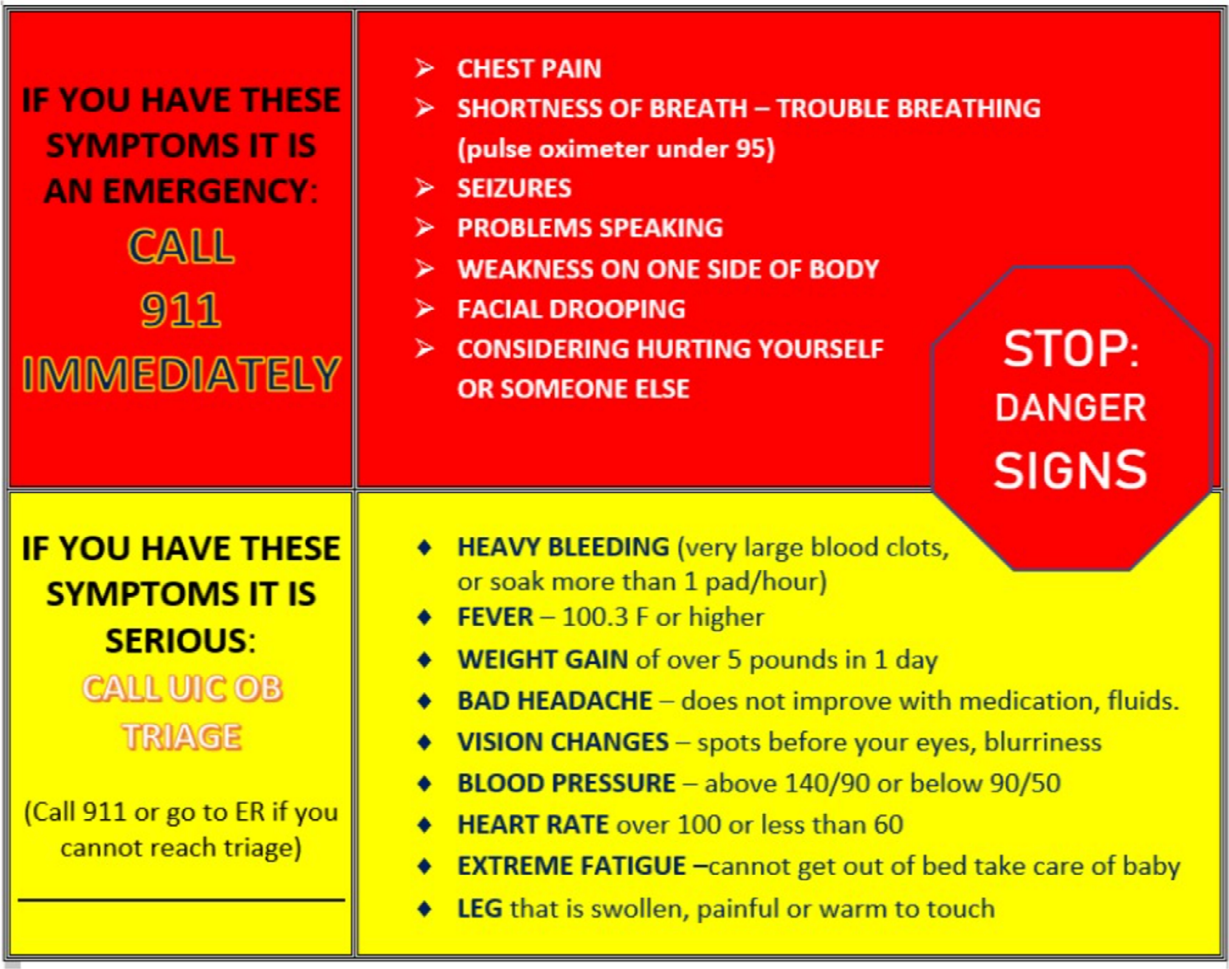
Postbirth Warning Signs Card An example of the postpartum danger signs and instructions given to participants and their families. Note: The warning signs card provided to participants included the OB triage phone number on one side, with the principal investigator and study coordinators’ contact numbers listed on the other side. Abbreviations: ER, emergency room; OB, Obstetrics; UIC, University of Illinois Chicago.

Black pregnant women were recruited and consented between 36 to 40 weeks’ gestation. On the first or second day postpartum, a researcher visited the participant in the hospital, reviewed study procedures and danger signs for maternal morbidity and mortality (Figure 1), and provided all study supplies. After birth, women were categorized by risk status (high or low).^19^ Low‐risk women did not have high BP, cardiac disease, diabetes, cesarean births, or other health complications during the current pregnancy. High‐risk women experienced one or more of the following morbidities during the current pregnancy: cesarean birth, chronic hypertension, hypertensive disorder of pregnancy, cardiac disease, heart failure, or thromboembolism.^19^ All women were given home monitoring equipment to track their health and recovery, including an automatic BP cuff, finger pulse oximeter (Healthsmart Intl, Waukegan, Illinois), a digital precision bathroom scale (Taylor, Oakbrook, Illinois), and a folder containing tables to log their data. They were instructed to record twice‐daily vital signs and daily weight. Daily weight was obtained to rule out the impending risk of heart failure, defined as at least 5 lb of weight gain in one day. Women were given 2 wearable investigative patches (one for daily use, the other as a backup) with charging ports to wear twice daily for 10 minutes. All participants were also given a Samsung Galaxy Tablet (Samsung, Seoul, South Korea) to upload and transmit the wearable patch data daily for analysis and to facilitate communication with the team.

On the day after hospital discharge, participants were called to review postpartum danger signs and to review study procedures. Based on participant preference, weekly telephone calls or Zoom meetings (Zoom Video Communications, Inc., San Jose, California) were scheduled to go over collected participant data. During the weekly calls, postpartum women were contacted to determine whether they had postpartum complications and to provide support and answer their questions. Participants were also advised to contact study personnel if any measurements fell outside normal limits (HR >100 or <60; respiration <12 or >22/min, temperature >100.3 °F; weight gain of >5 lb/day; BP <90/50 or >140/90 mm Hg; blood oxygen saturation [SpO_2_] <95%) and to call an emergency number if any readings were in the “danger” category outlined on a warning signs card (Figure 1). At the end of the 4 weeks, women were sent measures of feasibility, acceptability,^20^ appropriateness,^20^ and usability to complete on their own. All survey data were prospectively collected and stored on REDCap, a secure electronic database.^21^, ^22^

### Instruments

#### Wearable Patch

**Figure 2:**
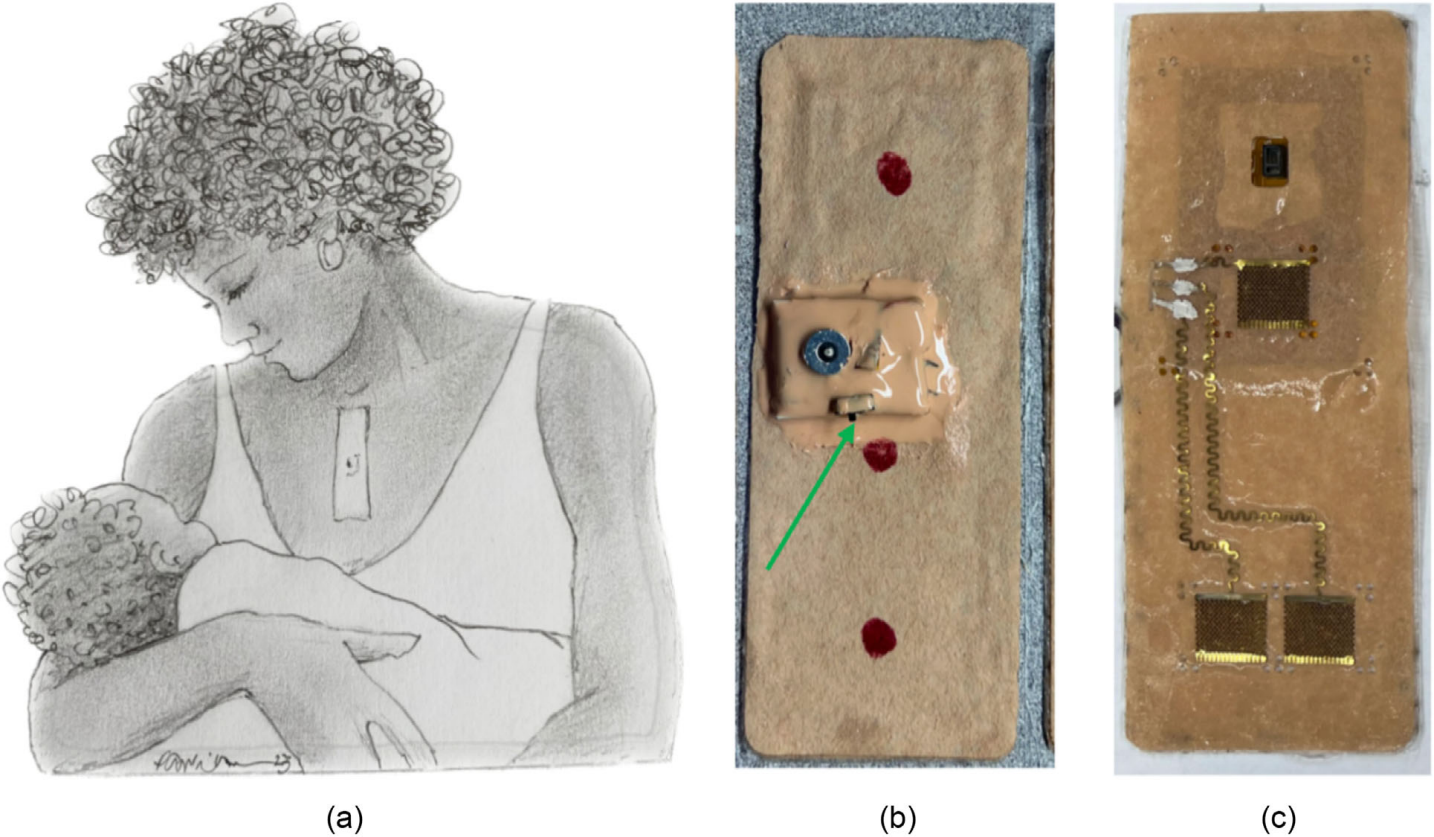
Prototype Patch and Placement on Chest (a) Image of a Black woman wearing the wearable patch with sensors on the chest. (b) Front of prototype wearable patch with on and off switch (green arrow) and red dots to indicate that the participant needed to apply pressure to ensure sensor contact with maternal skin. (c) The back side of the patch, showing the sensors. Image courtesy of Pavlina Vagoun‐Gutierrez.

**Figure 3:**
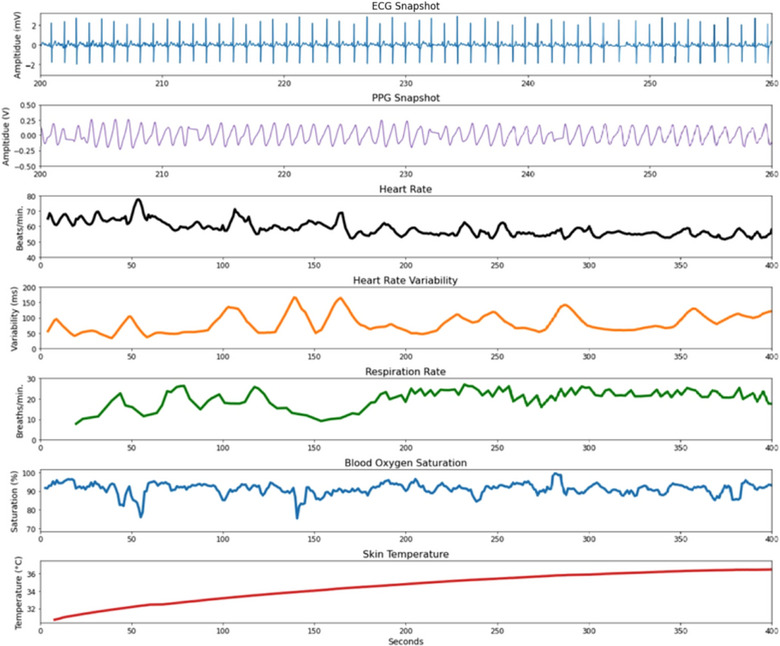
Patch Outputs on Tablet Wearable patch outputs of one woman in the study. Electrocardiogram (ECG): records the electrical signals of the heart. Photoplethysmography (PPG) snapshot: used to evaluate the strength and adequacy of the PPG signal. Heart rate: frequency of the heartbeat. Heart rate variability in milliseconds: examines neural mechanisms that modulate heart rate. Respiratory rate: the number of breaths a person takes per minute. Blood oxygen saturation: the percentage of oxygen‐bound hemoglobin in the blood. Skin temperature: the temperature of the body's outermost surface.

To characterize cardiovascular health, a flexible, wearable patch was designed to collect ECG and photoplethysmography (PPG) data with skin‐conformal electrodes and an integrated optical sensor. Figure 2 displays images of the wearable patch and where the patch was placed on the woman's chest. The PPG sensor is an optical technique that measures blood volume variations.^17^, ^23^ PPG signals were characterized for accuracy by evaluating the strength and adequacy of the signal.^12^ Within the PPG signals, other physiologic data can be extracted, including skin temperature, HR, HRV, respiratory rate (RR), and SpO_2_.^24^ HR, SpO_2_, and temperature were displayed to the user on the tablet for real‐time self‐monitoring. At the end of the measurement session, women uploaded the waveform files to a cloud server for processing. HR, HRV, RR, and SpO_2_ were calculated for participants and shared with the clinical team to review out‐of‐bound metrics. PPG calculated SpO_2_ data were then validated with self‐reported finger pulse oximeter readings. Figure 3 shows an example of the patch outputs from a woman in the study. The ECG and PPG signals were filtered to remove baseline wander and high‐frequency noise, and HR, HRV, RR, and SpO_2_ were calculated using standard techniques.^12^ The wearable patch was smaller than a credit card, rechargeable, reusable, Bluetooth‐enabled, and waterproof. It was designed to collect medical‐grade, high‐quality physiologic data.

Darker skin tones, inherently reflecting less visible and near‐visible wavelengths, present challenges to optical methods such as PPG that can be addressed (although not entirely solved) by optimizing the device design for maximum sensor contact with the women's skin. The patch achieves this through a combination of skin‐safe medical adhesives, flexible circuitry, and a custom internal spring mechanism to apply pressure on the PPG while maintaining skin conformality. Notably, the wearable PPG signal in the patch does so without sacrificing convenience, for instance, allowing women to have painted or fake nails, unlike the conventional finger pulse oximetry. In waveform evaluation, the patch design demonstrates the ability of the onboard PPG sensor to collect this signal from the sternum and produce SpO_2_ values that agree with those provided simultaneously by the pulse oximetry device.^12^

#### HR Variability

HRV parameters were used to examine neural mechanisms that modulate HR. The R to R interval, SD of normal sinus beats, and root mean square of successive differences were calculated. Our frequency band analysis focused only on the low‐ and high‐frequency oscillations in the HRV signal (defined as oscillations in the range of 0.4‐0.15 Hz vs 0.15‐0.4 Hz, respectively). The SD of normal sinus beats values greater than 100 ms are generally interpreted to reflect a healthier cardiovascular system. The SD of normal sinus beats less than 50 ms has been associated with an elevated risk for cardiovascular mortality. Unlike the SD of normal sinus beats (which reflects both sympathetic and parasympathetic modulation of HR), the root mean square of successive differences between normal heartbeats reflects the vagal nerve signals (parasympathetic) that regulate electrical conduction through the atrial and ventricular myocardium. Both the SD of normal sinus beats and root mean square of successive differences between normal heartbeats provide measures of the degree of variability in the heart rhythm.^17^, ^25^

#### Implementation Outcome Measures

Feasibility is defined as how easy it is to use a new intervention in a specific setting.^26^ It was assessed using the 4‐item Feasibility of Intervention Measure (FIM), which has a Cronbach's α of 0.88.^20^, ^26^ The score range was 4 to 20, with 4 being the least feasible and 20 being the most feasible (see Supporting Information: Appendix S2 for survey questions).

Acceptability refers to how much a participant likes the intervention.^26^ It was assessed with the 4‐item Acceptability of Intervention Measure (AIM), α = 0.80.^26^ The score range was 4 to 20, with 4 being the least acceptable and 20 being the most acceptable (see Supporting Information: Appendix S2 for survey questions).

Appropriateness refers to how well the new intervention fits the needs of the users and how well it addresses the problem it aims to solve.^26^ It was assessed using the 4‐item Intervention Appropriateness Measure (IAM), α = 0.73.^26^ The score range was 4 to 20, with 4 being the least appropriate and 20 being the most appropriate (see Supporting Information: Appendix S2 for survey questions).

The system usability scale (SUS)^27^ measures the intervention software's user‐friendliness and ease of use. This study assessed usability with the SUS, a 10‐item scale adapted to evaluate how easy it was to use the wearable patch. Scores ranged from 0 to 100, with 0 indicating no usability and 100 indicating the most usability (see Supporting Information: Appendix S2 for survey questions).

### Data Analysis

Descriptive statistics were calculated for all research variables using Excel (Microsoft Corporation, Excel for Mac 2022, Version 16.67, Redmond, Washington). Significant differences between the high‐risk and low‐risk groups were not calculated because this was a feasibility study with a small sample size.

## RESULTS

Twenty women consented to the study, but one withdrew before any data were collected. One woman did not have access to the internet upon returning home from the hospital and could not complete the patch portion of the study. Having internet access at home was a qualifier for the study, but the woman reported losing internet access due to financial challenges. All women had darkly pigmented skin tones. Women's characteristics are displayed in Table 1. Table 2 displays the low‐ and high‐risk maternal and newborn outcomes.

**Table 1:** Characteristics of 20 Women Enrolled in the Study by Group

Characteristics	Low‐Risk Group (n = 7)	High‐Risk Group (n = 13)
**Participant age**, **mean (SD); (range), y**	24.0 (3.4); (18‐28)	31.4 (5.4); (23‐39)
**Height, mean (SD); (range), inches**	63.9 (1.6); (62‐66)	65.1 (2.3); (62‐69)
**Prepregnancy weight, mean** **(SD); (range), lb**	223 (55.9); (167‐302)	221.3 (75.4); (147‐369)
**Prepregnancy BMI, mean (SD); (range)**	38.7 (10.7); (28.1‐53.5)	36.8 (14.0); (23.7‐67.5)
**Cardiac disease, n (%)**	0 (0)	2 (15)
**Anemia, n (%)**	3 (43)	7 (54)
**Chronic hypertension, n (%)**	0 (0)	5 (38)
**Lung/breathing issues, n (%)**	2 (29)	3 (23)
**Smoking during pregnancy, n (%)**	0 (0)	3 (23)
**Prior pregnancy, n (%)**		
None	3 (43)	2 (15)
1	0 (0)	1 (8)
2 or more	4 (57)	10 (77)
**Prior pregnancy outcomes, n (%)**		
Full‐term	6 (67)	13 (45)
sPTB	0 (0)	2 (7)
Induced preterm	1 (11)	1 (3)
Miscarriage or abortion	2 (22)	12 (41)
Stillborn	0 (0)	1 (3)
**History of pregnancy complications, n (%)**		
None	5 (63)	9 (60)
Preeclampsia	1 (13)	2 (13)
Abruption	0 (0)	1 (7)
Fetal distress	0 (0)	1 (7)
Short cervix	0 (0)	2 (13)
Other	2 (25)	0 (0)
**Fetal growth concern (current pregnancy), n (%)**	2 (29)	3 (23)
**High BP during (current pregnancy), n (%)**	0 (0)	7 (54)
**Gestational diabetes (current pregnancy), n (%)**	0 (0)	2 (15)
**Thromboembolism (current pregnancy), n (%)**	0 (0)	2 (15)

Abbreviations: BMI, body mass index; BP, blood pressure; sPTB, spontaneous preterm birth.

Source: Some percentages are based on occurances, as participants may have multiple outcomes.

**Table 2:** Pregnancy and Newborn Outcomes of 19 Women Who Received 4 Weeks of Postpartum Remote Monitoring^a^

Outcomes	Low‐Risk Group (n = 7)	High‐Risk Group (n = 12)
**Newborn birthweight, mean (SD), g**	2798 (316)	2836 (1242)
**Gestational age at birth, mean (SD), wk**	39 (1)	37 (5)
**Newborn sex, n (%)**		
Male	4 (57)	7 (58)
Female	3 (43)	5 (42)
**Mode of birth, n**		
Vaginal	7 (100)	6 (50)
Cesarean	0 (0)	6 (50)
**Self‐reported vital signs**		
**Maternal weight, mean (SD), lb**		
Postbirth	218 (55)	218 (79)
Week 1	213 (53)	212 (77)
Week 2	209 (52)	209 (70)
Week 3	207 (52)	206 (71)
Week 4	211 (53)	208 (72)
**Finger pulse oximetry, range, %**		
Postbirth	97‐99	94‐99
Week 1	91‐100	86‐100
Week 2	90‐100	85‐100
Week 3	94‐100	95‐100
Week 4	92‐100	94‐100
**Self‐reported: Wearable patch recordings (14 possible weekly), n (%)**		
Week 1	7 (50)	7 (50)
Week 2	7 (50)	10 (71)
Week 3	6 (43)	8 (57)
Week 4	6 (43)	9 (64)
**Usability of wearable patch score,** ^b^ **mean**		
Score at final visit	78	82
**Overall study scores for acceptability, appropriateness, feasibility,** ^c^ **mean**		
Acceptability	18.1	18.8
Appropriateness	18.1	18.7
Feasibility	17.7	18.8

a 20 women consented to the study, but 1 high‐risk woman dropped out of the study after she consented.

b Scale: 0 = not useable, 100 = most useable.

c Scale: 0 = least, 20 = most.

### Study Feasibility, Acceptability, and Appropriateness

Using the FIM,^20^ AIM,^20^ and IAM^20^ scores, women in the study rated all items related to the feasibility, acceptability, and appropriateness of remote physiologic monitoring highly. Regarding feasibility, all women agreed that remote physiologic monitoring seemed implementable, doable, and easy to use. Additionally, 18 of 19 women agreed that remote physiologic monitoring seemed possible, whereas 1 of 19 neither agreed nor disagreed. Concerning acceptability, all women agreed that remote physiologic monitoring met their approval, found it appealing, liked it, and welcomed its use. For intervention appropriateness, 18 of 19 women agreed that remote physiologic monitoring seemed fitting, whereas 1 of 19 neither agreed nor disagreed. All women agreed that remote physiologic monitoring seemed suitable, applicable, and like a good match (Table 2).

### Usability of Wearable Sensors Physiologic Monitoring

The usability of the wearable patch with sensors was rated high using the SUS^26^ (Table 2). Women reported feeling confident in their use of the wearable patch and that it was easy to use. When evaluating their desire to use the patch frequently, based on a subscale of SUS, 2 of 18 responders disagreed, and 16 of 18 agreed. When evaluating whether participants thought the patch was easy to use, 1 of 18 responders disagreed, 1 of 18 neither agreed nor disagreed, and 16 of 18 agreed. When evaluating whether participants found the wearable patch cumbersome, 11 of 18 participants disagreed, 3 of 18 neither agreed nor disagreed, and 4 of 18 agreed.

### Wearable Physiologic Sensor Measures in the Postpartum Period

Data in Table 3 reflect HRV values acquired and averaged throughout the study. HRV data were not available for 2 women in the study (one participant did not collect PPG data due to lack of internet access, and one did not have interpretable PPG data). HRV was calculated from ECG data. All women's HRs were within normal limits (60‐100 beats per minute). Women in the high‐risk group had a mean HR of approximately 5 beats per minute faster than those in the low‐risk group. The R to R interval of the ECG, the SD of normal sinus beats, and the root mean square of successive differences were similar between both groups. Considering the brief duration of the ECG recordings, our frequency band analysis focused only on the low‐frequency and high‐frequency oscillations in the HRV signal (low‐frequency and high‐frequency defined as oscillations in the range of 0.04‐0.15 Hz vs 0.15‐0.4 Hz, respectively). Spectral analysis revealed similar low‐frequency and high‐frequency power between the groups (Table 3).

**Table 3:** Heart Rate Variability Metric Results^a^

	Time‐Domain HRV Metrics				Frequency‐Domain HRV Metrics		
Group	Heart Rate, Beats/min Mean (SD)	RRI, ms Mean (SD)	SDNN, ms Mean (SD)	RMSSD, ms Mean (SD)	LF Power, ms^2^ Mean (SD)	HF Power, ms^2^ Mean (SD)	LF:HF Ratio Mean (SD)
All participants (n = 17)^b^	85.7 (8.2)	240.4 (197.7)	477.4 (330.1)	0.43 (0.88)	0.05 (0.06)	0.06 (0.07)	1.57 (0.99)
Low‐risk (n = 6)	82.2 (6.3)	183.6 (176.6)	392.1 (322.1)	0.29 (0.64)	0.04 (0.05)	0.04 (0.05)	1.49 (1.17)
High‐risk (n = 11)	87.2 (8.9)	263.5 (201.4)	512.2 (327.5)	0.49 (0.96)	0.05 (0.06)	0.06 (0.07)	1.46 (0.90)

Abbreviations: HF, high‐frequency; HRV, heart rate variability; LF, low‐frequency; RMSSD, root mean square of successive differences; RRI, R to R interval; SDNN, SD of normal sinus beats.

a Each heart rate variability metric was averaged for every participant's data throughout the 28‐day study to calculate group means.

b HRV data were not available for 1 low‐risk and 1 high‐risk woman.

### Self‐Reported Physiologic Measures in the Postpartum Period

**Figure 4:**
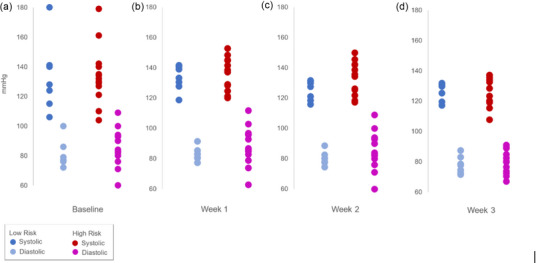
Blood Pressure Data Illustrates the systolic and diastolic readings for each participant, showing one baseline reading (a) and their average weekly pressures (b‐d). Note: Some women in both groups had high mean readings during weeks 1 and 2 (ie, >140 mm Hg systolic or >90 mm Hg diastolic).

Self‐reported physiologic measures (BP, pulse oximetry, and weight) were collected to quantify the participant's physiologic functions.^28^ Out of 14 possible weekly patch recordings and transmissions, the low‐risk group completed 6 to 7 (43%‐50%) of the recordings and transmissions, and the high‐risk group completed 7 to 10 (50%‐71%). The average weekly participant‐recorded vital signs are listed in Table 2. Figure 4 illustrates each woman's systolic and diastolic readings, showing one baseline reading (panel A) and their average weekly pressures (panels B‐E). Mean systolic and diastolic BPs did not differ between the low‐ and high‐risk groups. Importantly, some women in both groups had high mean BP readings during weeks 1 and 2 (ie, >140 mm Hg systolic or >90 mm Hg diastolic).

The low‐risk and high‐risk groups completed 88% of the BP readings. BP readings were the highest at baseline and at week 1 (Figure 4) and generally decreased by week 4. Fifteen women were advised to discuss their recorded BPs with a health care provider due to elevated readings. Seven women did not report addressing the issue with their providers. Three women visited a health care provider but did not receive medical interventions for their high BP. Five women received prescription medication or had their dosage adjusted. The average weekly recorded weights are listed in Table 2. None of the women had more than a 5‐lb daily weight gain. Both groups completed and recorded at least 85% of the self‐reported pulse oximetry measurements and weights over the 4‐week monitoring period. Several women had false or painted nails and had abnormal pulse oximetry readings, whereas all their other vitals were normal, suggesting pulse oximetry measurement error.

## DISCUSSION

### What We Learned From Our Study

In general, participants rated the acceptability,^20^ appropriateness,^20^ feasibility,^20^ and useability^20^ of remote home monitoring with wearable sensors as highly favorable. However, the low‐risk group only completed 6 to 7 of 14 (43%‐50%) weekly wearable patch recordings, whereas the high‐risk group completed 7 to 10 of 14 (50%‐71%) (Table 2). The reasons for the discrepancy between survey scores and patch use remain unclear. One study^29^ suggested that healthy women may perceive postpartum care as lacking value for themselves, which could lead to lower prioritization of tasks like recording patch data. Additionally, considering that 7 of 18 women reported the patch as cumbersome or were indifferent about its usability, the use of a more conveniently worn device, such as a PPG watch, that does not have to be taken off and could send the data without being processed manually could enhance adherence.^30^

### Clinical Implications

Remote monitoring may be especially important because rates of follow‐up care for underserved Black women are low.^31^ Moreover, health care providers do not consistently identify and treat postpartum risk factors, especially hypertension.^32^ There is a lot of clinical and research interest in developing remote monitoring tools for pregnancy to prevent complications.^33^, ^34^, ^35^ Our novel approaches, along with those of others,^33^, ^34^, ^35^ can be implemented outside of the standard postpartum office visits and may help prevent postpartum maternal morbidity and potential mortality.

Some of the women recorded systolic BPs greater than 140 mm Hg and/or diastolic BPs greater than 90 mm Hg within the first 2 weeks after giving birth. More than half of the women did not have a prior history of hypertension during the present or previous pregnancy.^36^, ^37^ A study of postpartum readmissions found that women who did not have chronic or gestational hypertension were at significant risk of readmission due to de novo onset of hypertension.^38^ A key aspect of pregnancy‐associated cardiovascular complications is that they frequently present postpartum.^39^ Up to 14% to 16% of overall sudden maternal morbidity presents de novo following discharge.^39^ Early intervention with therapies that protect cardiovascular and postpartum health may prevent long‐term cardiovascular complications as well as maternal death.^1^, ^3^, ^4^, ^40^ Monitoring and ECG analysis offer the potential of an early warning system to prevent cardiovascular complications in the postpartum period.

Because 7 women with high BP did not seek treatment or adhere to the study protocol, other behavioral factors may be important in motivating women to act upon their results. A qualitative study^41^ reported that patients seek family advice, and families play an important role in medication adherence for cardiovascular disease risk factors. The patient's need for self‐efficacy in deciding when to take medications was also a reported factor.^41^ Because postpartum care is so fragmented, women and their health care providers may not have a strong enough relationship to promote adherence. Other factors, such as the desire to follow the advice of family and influential friends, as well as social/economic factors like high copays, insurance issues, and transportation, may play a role.^41^ Identifying physiologic danger signs may be the first step in reducing maternal morbidity. More research is needed to identify interventions that are acceptable to Black postpartum women to achieve the desired clinical goals.

Our results lead us to question the utility of traditional risk factor categories for Black women, particularly the effectiveness of classifying them as high‐risk or low‐risk in predicting poor cardiovascular health outcomes after childbirth. We found similar HRV profiles in the low‐risk and the high‐risk groups. Our findings indicated greater HRV, along with a decreased lower‐frequency to high‐frequency ratio in the present study, suggesting the possibility of a higher parasympathetic modulation of the HR in the high‐risk group, usually a positive indicator of cardiovascular health. Because we found more favorable HRV profiles in the high‐risk group, our findings suggest that the low‐risk and high‐risk designations may not reflect useful or valid categories to determine cardiovascular health in Black postpartum women.

### Research Implications

With the ongoing advancements in wearable patch technology, remote monitoring can be completely automated, including the ability to notify health care providers of panic values. The Food and Drug Administration has not approved any of the commercially available health watches for use in clinical care. Our patch was deemed to be an investigational device by our IRB. It is well known that PPG sensors used in this research and most finger pulse oximeters can yield inaccurate results due to motion artifact, poor signal, poor blood flow, stiff vessels, and diverse skin types.^42^ In our study, the PPG signal was evaluated for adequacy, and the results were comparable to the self‐reported SpO_2_ measures. It is possible that pulse oximetry devices could be developed for different populations based on skin tone and clinical conditions.^42^ Known challenges of conventional pulse oximeters with darker skin tones can be reduced but not solved with careful device form factor design. Our patch was deliberately designed to be conformal and flexible for maximum signal‐to‐noise ratio (SNR), and a passive force mechanism was additionally developed to apply the recently reported optimal PPG force (1 newton) at the sternum. These choices were valuable but not sufficient for capturing PPG at the sternum for women with darker skin tone. As described in our accompanying publication^12^ on device design, high‐quality PPG (>16 dB SNR) was captured from the device for multiple patients. PPG inaccuracies of pulse oximetry in dark‐pigmented individuals can occur because darker‐pigmented skin contains more melanin, absorbing more green light than lighter skin.^42^ PPG wavelength signals could use multiple colors of light that penetrate skin pigment and materials.^16^, ^43^ Consistent with other reports,^44^, ^45^ we also found that in participants with darkly painted fingernails, there were abnormal pulse oximetry results when all other vital signs were normal.

### Strengths and Limitations

A limitation of this study is social desirability bias. To minimize this in future research, we will ensure surveys are collected anonymously and deidentified. Additionally, we plan to incorporate scales designed to measure social desirability bias within our survey, allowing us to control for it in our analysis and future study designs. A strength of our study was the number of vital sign recordings over a 4‐week period. Our findings need to be examined in a future fully powered study to understand how altered HRV metrics and BP changes could be used to predict maternal outcomes. We did not measure HRV before or during pregnancy. Sarhaddi et al^15^ studied HRV during and after pregnancy and found a reduction of HRV in the second trimester and in women who developed preeclampsia and gestational diabetes.^46^, ^47^, ^48^, ^49^ HRV parameters are not generally used in routine clinical care; however, they might be useful as a biomarker for increased risk of cardiovascular complications postpartum. Future innovations must develop the technology and system requirements to automate remote monitoring and care. In the era of personalized health care, valid technologies for specific populations need to be developed and tested.

## CONCLUSION

In our study, remote monitoring in the first 4 weeks postpartum successfully identified women at risk for complications. Moreover, women were receptive to remote monitoring and rated it as feasible, acceptable, useable, and appropriate. Further larger clinical trials will need to be performed to best identify which patients derive benefit and to optimize device use so that it requires minimal user input and monitoring is seamless. The postpartum period is characterized by decreased health surveillance and increased morbidity and mortality. Remote monitoring has the potential to increase surveillance and thereby reduce complications for Black postpartum women.

## CONFLICT OF INTEREST

The authors have no conflicts of interest to disclose.

## Supporting information




**Appendix S2**. Survey questions
