# Supplementary material for: Feasibility of Remote Intensive Monitoring: A Novel Approach to Reduce Black Postpartum Maternal Cardiovascular Complications
Source: J Midwifery Womens Health. 2025 Feb 26;70(4):549–59. doi: 10.1111/jmwh.13743 (PMC12365727; doi:10.1111/jmwh.13743)
Supplement: Supplementary file 1 — Appendix S2. Survey questions [file JMWH-70-549-s001.docx]

Appendix S2. Adapted survey questions from the Acceptability of Intervention Measure (AIM), Intervention Appropriateness Measure (IAM), and Feasibility of Intervention Measure (FIM)

**Feasibility of Intervention Measure (FIM)**

|  | Completely disagree | Disagree | Neither agree nor disagree | Agree | Completely agree |
| --- | --- | --- | --- | --- | --- |
| 1. Remote physiological monitoring seems implementable. | ➀ | ➁ | ➂ | ➃ | ➄ |
| 2. Remote physiological monitoring seems possible. | ➀ | ➁ | ➂ | ➃ | ➄ |
| 3. Remote physiological monitoring seems doable. | ➀ | ➁ | ➂ | ➃ | ➄ |
| 4. Remote physiological monitoring seems easy to use. | ➀ | ➁ | ➂ | ➃ | ➄ |

**Intervention Appropriateness Measure (IAM)**

|  | Completely disagree | Disagree | Neither agree nor disagree | Agree | Completely agree |
| --- | --- | --- | --- | --- | --- |
| 1. Remote physiological monitoring seems fitting. | ➀ | ➁ | ➂ | ➃ | ➄ |
| 2. Remote physiological monitoring seems suitable. | ➀ | ➁ | ➂ | ➃ | ➄ |
| 3. Remote physiological monitoring seems applicable. | ➀ | ➁ | ➂ | ➃ | ➄ |
| 4. Remote physiological monitoring seems like a good match. | ➀ | ➁ | ➂ | ➃ | ➄ |

**Acceptability of Intervention Measure (AIM)**

|  | Completely disagree | Disagree | Neither agree nor disagree | Agree | Completely agree |
| --- | --- | --- | --- | --- | --- |
| 1. Remote physiological monitoring meets my approval. | ➀ | ➁ | ➂ | ➃ | ➄ |
| 2. Remote physiological monitoring is appealing to me. | ➀ | ➁ | ➂ | ➃ | ➄ |
| 3. I like remote physiological monitoring. | ➀ | ➁ | ➂ | ➃ | ➄ |
| 4. I welcome remote physiological monitoring | ➀ | ➁ | ➂ | ➃ | ➄ |

**Appendix S2.** Adapted survey questions from the System Usability Scale (SUS)

**System Usability Scale (SUS)**

|  | Strongly disagree | Disagree | Neither agree nor disagree | Agree | Strongly agree |
| --- | --- | --- | --- | --- | --- |
| 1. I think that I would like to use the wearable patch frequently. | ➀ | ➁ | ➂ | ➃ | ➄ |
| 2. I found the wearable patch unnecessarily complex. | ➀ | ➁ | ➂ | ➃ | ➄ |
| 3. I thought the wearable patch was easy to use. | ➀ | ➁ | ➂ | ➃ | ➄ |
| 4. I think that I would need the support of a technical person to be able to use this wearable patch. | ➀ | ➁ | ➂ | ➃ | ➄ |
| 5. I found the various functions in this wearable patch were well integrated. | ➀ | ➁ | ➂ | ➃ | ➄ |
| 6. I thought there was too much inconsistency in this wearable patch. | ➀ | ➁ | ➂ | ➃ | ➄ |
| 7. I would imagine that most people would learn to use this wearable patch very quickly. | ➀ | ➁ | ➂ | ➃ | ➄ |
| 8. I found the wearable patch very cumbersome to use. | ➀ | ➁ | ➂ | ➃ | ➄ |
| 9. I felt very confident using the wearable patch. | ➀ | ➁ | ➂ | ➃ | ➄ |
| 10. I needed to learn a lot of things before I could get going with this wearable patch. | ➀ | ➁ | ➂ | ➃ | ➄ |
